# Case Report: Area of Focus of Myocardial Infarction With Non-obstructive Coronary Arteries in Eosinophilic Granulomatosis With Polyangiitis

**DOI:** 10.3389/fcvm.2021.731897

**Published:** 2021-11-12

**Authors:** Xiaoxian Cui, Yang Peng, Jun Liu, Yugang Dong, Zexuan Wu, Yili Chen

**Affiliations:** ^1^Respiratory Department, The Eighth Affiliated Hospital, Sun Yat-sen University, Shenzhen, China; ^2^Department of Radiology, The First Affiliated Hospital, Sun Yat-sen University, Guangzhou, China; ^3^Department of Cardiology, The First Affiliated Hospital, Sun Yat-sen University, Guangzhou, China; ^4^National Health Commission (NHC) Key Laboratory of Assisted Circulation, Sun Yat-sen University, Guangzhou, China

**Keywords:** EGPA, MINOCA, case report, cardiac magnetic resonance, STEMI

## Abstract

**Background:** Eosinophilic granulomatosis with polyangitis manifested as myocardial infarction with non-obstructed coronary arteries (MINOCA) is rarely reported.

**Case:** We report a 43-year-old male patient without any cardiovascular risk factors presenting with acute chest pain. Electrocardiogram was suggestive of acute anterior and inferior myocardial infarction. MINOCA was confirmed based on significant elevated cardiac troponin and normal coronary arteries. Cardiac magnetic resonance (CMR) imaging revealed extended late gadolinium enhancement (LGE). Further diagnosis of eosinophilic granulomatosis with polyangitis (EGPA) was based on clinical manifestations and auxiliary examination. Subsequent immunosuppressive therapy led to regression of symptoms and significant resolution of LGE on CMR.

**Conclusion:** Our case highlights that EGPA can be a rare cause of MINOCA. CMR is useful for differentiation diagnosis and evaluation of cardiac involvement.

## Introduction

ST-elevation myocardial infarction usually occurs from plaque rupture, erosion, fissuring, or dissection, which results in an obstructing thrombus. However, in some circumstance, coronary angiography fails to reveal obstructive coronary arteries in patients clinically defined by criteria of ST-elevation myocardial infarction (STEMI) (1), and a clinically overt specific cause for the acute presentation is unclear, which is concluded as myocardial infarction with non-obstructive coronary arteries (MINOCA) (2). Eosinophilic granulomatosis with polyangitis (EGPA) is a rare, systemic, necrotizing in small- and medium-sized blood vessels vasculitis, which could cause coronaritis or MINOCA. In this case, we report a young man with clinical criteria for STEMI with normal coronary arteries, which was eventually confirmed as EGPA.

## Case Presentation

A 43-year-old male presented to the emergency department of our hospital with a 5-day history of typical chest tightness radiating to his back. He was previously fit except for a 3-year history of recurrent cough and fever between 37 and 38°C with a significant weight loss of ~7.5 kg. He was diagnosed as asthma and had been received regular Seretide inhaler (salmeterol and fluticasone propionated inhalation) therapy for 1 year.

**Figure 1:**
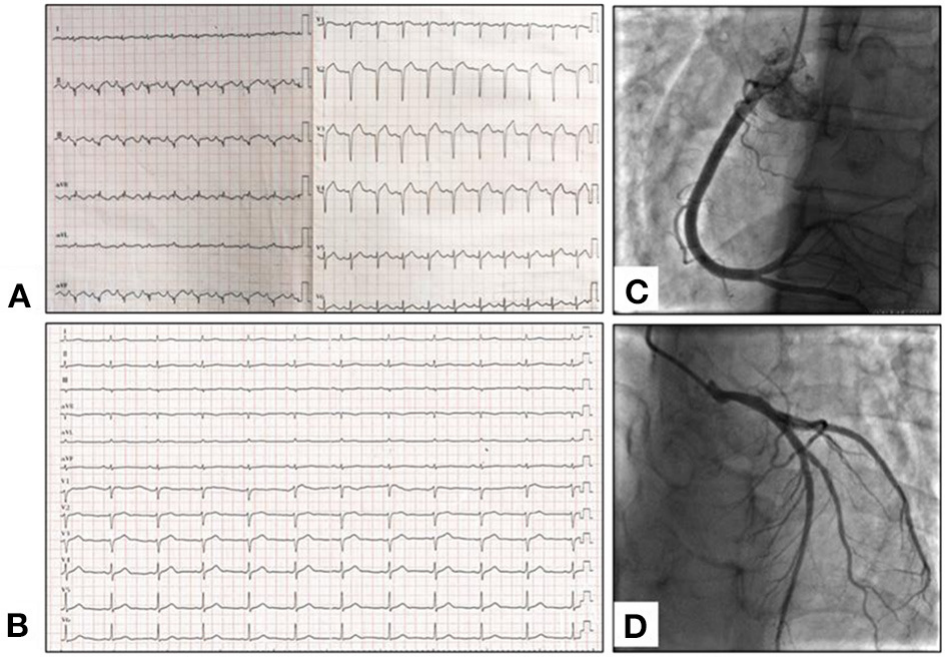
**(A)** Initial ECG at the emergency room showed poor R wave progression on precordial lead and QS wave on anterior and inferior leads. **(B)** Follow-up ECG after 3-month therapy showed increased R wave amplitude in inferior leads. **(C,D)** Coronary angiography demonstrates normal coronary arteries; **(C)** Right coronary artery. **(D)** Left coronary arteries.

**Figure 2:**
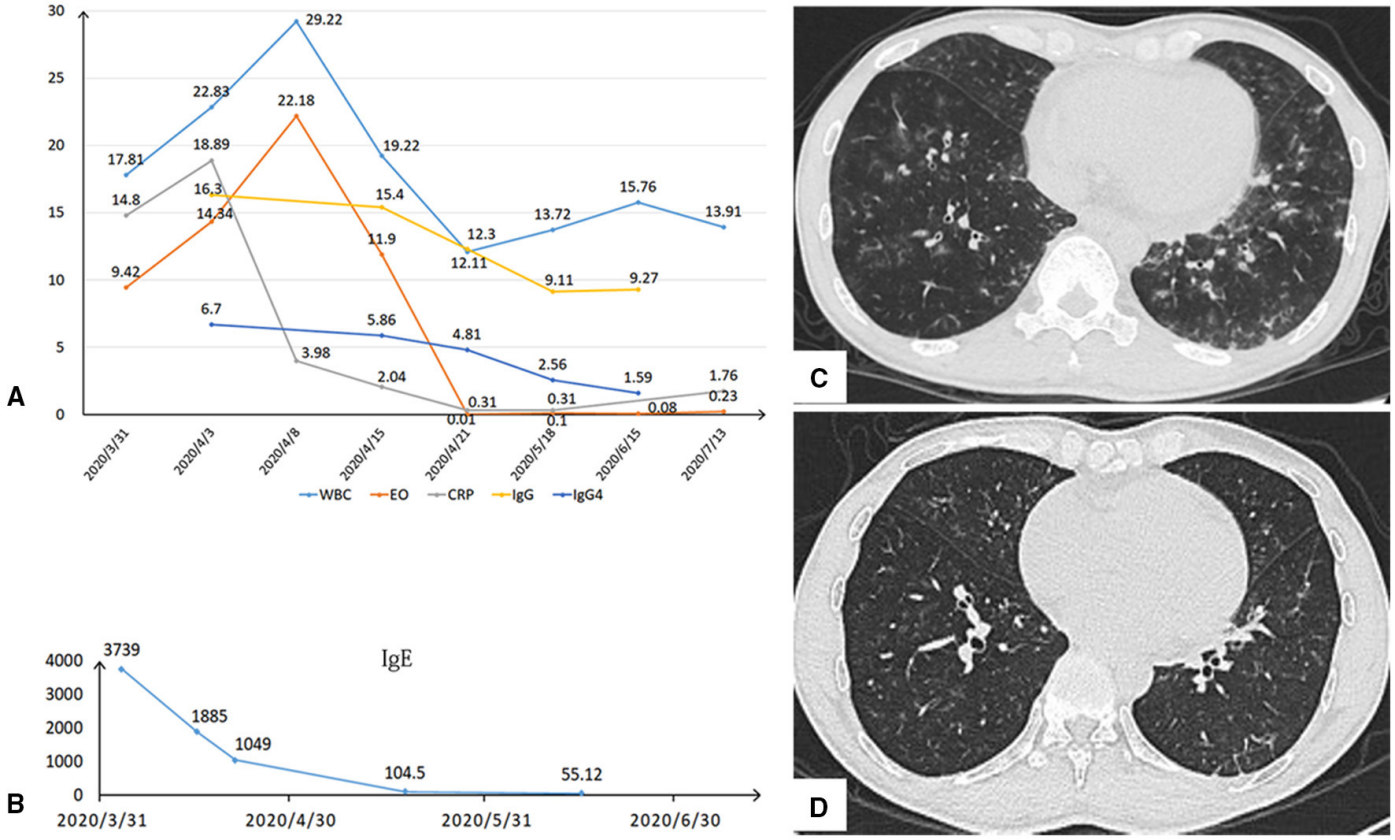
**(A,B)** After comprehensive treatment, the white blood cells (WBC), eosinophilia (EO), CRP, IgG, IgG4, and IgE responded rapidly and kept dropping down. **(C)** Chest CT indicated multiple lung infiltrates and small patches of ground-glass appearance in both lungs initially. **(D)** After half-a-month antibiotic therapy, the lung infiltration infiltrates obviously disappeared.

**Figure 3:**
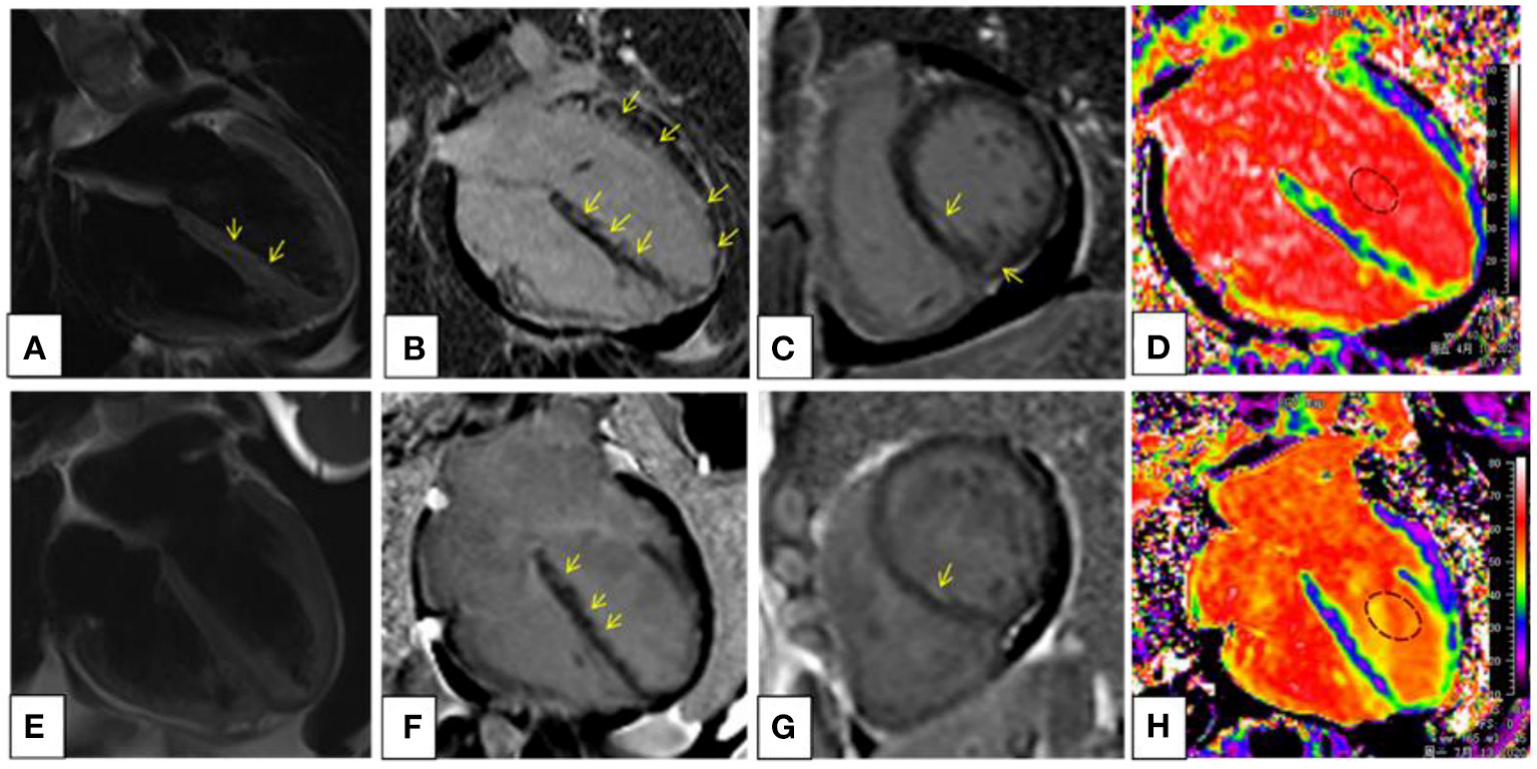
Cardiac magnetic resonance (CMR) imaging at the baseline **(A–D)** and after 3-month prednisone and immunosuppressant therapy **(E–H)**. T2-weighted image **(A)** demonstrated small patches of high-signal intensity in mid-wall and subendocardial regions of the interventricular septum, revealing myocardial edema. On the four-chambered **(B)** and basal short-axis **(C)** view of late gadolinium enhancement (LGE), multiple foci of high-signal intensity were scattered diffusely in mid-wall and subendocardial regions in a nonischemic pattern. An extracellular volume (ECV) map **(D)** showed heterogeneously elevated ECV fraction of the left ventricular myocardium and septum; the global ECV fraction was 34.6%. On posttreatment CMR, it showed complete remission of the myocardial edema **(E)** and significantly reduced LGE **(F,G)**. The global ECV fraction decreased to 29.6% **(H)**.

In the emergency department, his electrocardiogram (ECG) showed acute myocardial infarction (Figure 1A). Physical examination revealed sinus tachycardia of 116 beats per minute. His blood test revealed elevated creatine kinase-MB 38 U/L (normal 0.10–4.94 U/L) and high-sensitivity troponin-T 1.19 ng/ml (normal < 0.014). The total white blood cell count was 18.97 × 10^9^/L with marked eosinophilia 9.42 × 10^9^/L (normal 0.05–0.50 × 10^9^/L). Chest CT indicated multiple lung infiltrates and small patches of ground-glass appearance in both lungs (Figure 2C). Transthoracic echocardiogram (TTE) showed normal systolic and diastolic function (ejection fraction: 63%); however, increased echogenicity of endocardium and slightly reduced wall motion was detected in the basal segment of inferior wall. Thus, coronary artery disease could not be excluded. Considering it had been more than 12 h since the onset, coronary angiography (CAG) was not performed until Day 7, showing normal coronary arteries (Figures 1C,D). Thus, the diagnosis of MINOCA was confirmed. Cardiac magnetic resonance imaging (CMR) showed normal wall motion with inflammatory edema (Figure 3A), and late gadolinium enhancement (LGE) was found in multiple foci (Figures 3B–D).

Noticing his blood test of significant eosinophilia, we made further examinations to evaluate the underlying causes of MINOCA. The level of IgE was elevated to 3,739.00 IU/ml (normal 0–120 IU/ml). Anti-neutrophil cytoplasmic antibody was negative, while IgG and IgG4 were markedly elevated (19.10, 6.700 g/L, respectively normal: 10.13–15.13, ≤ 2.00 g/L, respectively) (Figures 2A,B). Bone marrow aspiration and biopsy were performed, and no parasites or hematological diseases were found. Furthermore, hematologic tumor cloning biomarkers were all negative.

In addition, paranasal sinus computed tomography (CT) revealed whole group sinusitis. Electromyography showed partial peripheral nerve damage in the upper and lower limbs and abnormal F waves. Since he fulfilled all the criteria of the six American College of Rheumatology (ACR) classification criteria for EGPA (3) and all other possible hypereosinophilic diseases were excluded, the diagnosis of EGPA-associated MINOCA was made.

After being diagnosed as EGPA, the patient started on intravenous methylprednisolone (40 mg/day), and his eosinophil count, CRP, IgG, IgG4, and IgE returned to normal within 1 week (Figures 2A,B), and the lung infiltrates obviously disappeared (Figure 2D). He was discharged from the hospital without any discomfort, and mycophenolate mofetil dispersible (0.75 mg QD) was added to methylprednisolone as maintenance. After 3 months, echocardiogram performed showed normal wall motion and cardiac function. ECG showed that the R wave amplitudes in lead II and avF increased significantly compared with those at the onset of MINOCA (Figure 1B). CMR showed regression of edema (Figure 3E) and significant resolutions of myocardial fibrosis (Figures 3F–H). Now, the maintenance dose of prednisone for this patient is gradually decreased to 17.5 mg per day, and the patient is still under follow-up.

## Discussion

Eosinophilic granulomatosis with polyangiitis is a systemic vasculitis with eosinophilia infiltrating small and medium-sized blood vessels in multiple organs. EGPA can be divided into three stages (4). In the prodromal stage, fever and a variety of respiratory symptoms often occur, and allergic rhinitis, sinusitis (multiple groups), and bronchial asthma often occur; the second phase is known as tissue eosinophil infiltration (including lung, myocardium, gastrointestinal tract, etc.) lasting for months to years; finally, the third stage presents as vasculitis, and multiple organ damage could occur. A diagnosis of EGPA is made when at least four out of the following criteria developed by the ACR in 1990 are presented; (1) asthma, (2) eosinophilia (>10% on differential white blood cell count), (3) mononeuropathy or polyneuropathy, (4) pulmonary infiltrates, (5) paranasal sinus abnormality, and (6) extravascular eosinophils. Myocardial abnormalities are found in more than 50% of patients with EGPA at autopsy (5). Cardiac involvement is usually the leading cause of death and is reported in 16–29% of patients with EGPA (6, 7). EGPA could involve any part of the heart and manifest as pericarditis, pericardial effusion, cardiac tamponade, congestive heart failure, or even myocardial infarction due to vasculitis of coronary vessels (7). Non-ST-elevation myocardial infarction (8–10) or STEMI with the etiologies of luminal stenosis or spasm throughout the coronary trees is reported in patients with EGPA in several cases (11–13). However, ST elevation with the etiology of myocarditis and myocardial fibrosis in EGPA has not been reported before. In our case, the patients with EGPA without any cardiovascular risk factors presented with ST elevation without coronary arteries stenosis, and, furthermore, CMR confirmed multifocal myocarditis and replacement fibrosis as the cause of MINOCA.

Currently, Cardiac magnetic resonance is the golden criterion for the identification and characterization of myocardial fibrosis associated with myocardial infarction and other nonischemic conditions (14). What is more, a direct association between the ischemic coronary artery and myocardial infarction can be established based on the precise localization of the infarcted area on CMR. And CMR is a clinically relevant noninvasive imaging modality for the assessment of patients presenting with MINOCA (15). The myocardial characteristics of CMR can be used to identify focal edema and fibrosis with STIR T2-weighted and late gadolinium enhancement (LGE) images (16) and can assess diffuse myocardial fibrosis with elevated extracellular volume (ECV) fraction. Myocardial fibrosis detected by LGE has been related to prognosis in various cardiac conditions, and the presence of LGE likely represents a marker of severity of cardiac disease in patients with EGPA (17). In our case, the long T2 signal in interventricular septum (yellow arrow) suggested inflammatory edema (Figure 3A), and LGE happened in multiple foci (Figures 3B,C), and ECV fraction was significantly elevated in the extended LGE region (Figure 3D). After 3-month prednisone and immunosuppressant therapy, follow-up CMR demonstrated the resolution of high-signal intensity on T2-weighted image, attenuation of LGE in multiple foci, and decreased global ECV fraction (Figures 3E–H). However, ECG presentation with ST-segment elevation seemed to be a poor prognostic marker in MINOCA (15). Although ECG showed significantly better after 3-month therapy, we do not know the overall survival time of the patient. Thus, long-term follow-up of CMR and ECG should be performed. In our case, CMR is useful for differentiation diagnosis and evaluation of cardiac involvement for patients with EGPA, and an excellent follow-up way to identify internal cardiac lesion while ECG and inspection result were negative.

According to a meta-analysis of 62 patients diagnosed with EGPA with cardiac involvement (18), EGPA may mimic acute coronary syndrome with nonspecific ST-T changes or, rarely, ST elevation on ECG, the etiologies of which may be coronary vasospasm, intracoronary thrombi, coronary artery stenotic lesions or coronary ectasia. In our case, no evidence of coronary vasospasm was revealed on CAG, and, considering the patient was in the acute phase of STEMI, we did not conduct a provocation test for coronary spasm. Besides, LGE distribution within the left ventricular wall was not consistent with coronary artery territory distribution, suggesting the cardiac involvement to be a nonischemic pattern. After corticosteroids and immunosuppressive therapy, LGE was significantly attenuated, which indicated that acute myocarditis caused by EGPA may be the etiology of MINOCA, possibly the acute eosinophilic myocarditis. There are also few reported cases of ST-segment or non-ST-segment elevation myocardial infarction as the presenting feature of patients with eosinophilic myocarditis (19–21). However, in our case, the patient refused endocardial biopsy that we could not make a definite diagnosis of acute eosinophilic myocarditis. Besides, the optical coherence tomography (OCT) may be valuable in the diagnosis of atherosclerotic etiology in individuals with MINOCA (22), yet, the patient did not perform the test, which may not identify whether plaque disruption was as an underlying cause of MINOCA in this case.

## Conclusion

This case emphasizes that cardiac involvement due to EGPA can be a rare cause of MINOCA. CMR can detect myocardial fibrosis through subendocardial or intramyocardial late gadolinium enhancement without invasion. Hence, CMR is useful for differentiation diagnosis and evaluation of cardiac involvement.

## Data Availability Statement

The original contributions presented in the study are included in the article/supplementary materials, further inquiries can be directed to the corresponding author/s.

## Ethics Statement

Written informed consent was obtained from the individual(s) for the publication of any potentially identifiable images or data included in this article.

## Author Contributions

XC performed the data analyses and wrote the manuscript. YP contributed to analysis and explained the image of the cardiac magnetic resonance. JL helped the clinical case analysis and directed the coronary angiography. YD was responsible for patient follow-up. ZW helped to perform the analysis with constructive discussions and editing of the original article. YC contributed to the presentation of the published work by those from the original research group, specifically critical review, commentary, or revision. All the authors contributed to the patient care, diagnosis, and treatment.

## Funding

This study was funded by the National Natural Science Foundation of China (No. 81800345) and Guangdong Natural Science Foundation (2018A030313448 and 2021A1515010433).

## Conflict of Interest

The authors declare that the research was conducted in the absence of any commercial or financial relationships that could be construed as a potential conflict of interest.

## Publisher's Note

All claims expressed in this article are solely those of the authors and do not necessarily represent those of their affiliated organizations, or those of the publisher, the editors and the reviewers. Any product that may be evaluated in this article, or claim that may be made by its manufacturer, is not guaranteed or endorsed by the publisher.
